# Non-trocar related major retroperitoneal bleeding during laparoscopic appendectomy

**DOI:** 10.1186/1749-7922-6-9

**Published:** 2011-03-22

**Authors:** Haytham MA Kaafarani, Julian D'Achille, Roger A Graham

**Affiliations:** 1 Department of Surgery, Tufts Medical Center, Boston, MA, USA; 2 Tufts University School of Medicine, Boston, MA, USA

## Abstract

Most of the reported vascular injuries in laparoscopic appendectomies occur during trocar or Veress needle insertions. As laparoscopy continues to evolve, it is essential that surgeons report unusual complications in an effort to raise awareness and guide management of any iatrogenic injury incurred during minimally-invasive procedures. We report the case of a patient who sustained a major non-trocar related retroperitoneal vascular injury during a routine LA.

## Introduction

Laparoscopic appendectomy (LA) has gained widespread acceptance in the last 2 decades. Multiple trials and meta-analyses have suggested that the laparoscopic approach offers patients a lower risk of surgical site infection, less postoperative pain, a shorter length of stay and earlier return to work when compared to open appendectomy (OA) [1-6]. Nonetheless, the same studies found higher rates of intra-abdominal abscesses and vascular injuries with LA. Most of the reported vascular injuries in laparoscopy occur during trocar or Veress needle insertions [7]. For patients over the age of 65, population-based studies have even suggested a lower mortality with LA [8]. As laparoscopy continues to evolve, it is essential that surgeons report unusual complications in an effort to raise awareness and guide management of any iatrogenic injury incurred during minimally-invasive procedures. We report the case of a patient who sustained a major non-trocar related retroperitoneal vascular injury during a routine LA.

## Case Report

**Figure 1:**
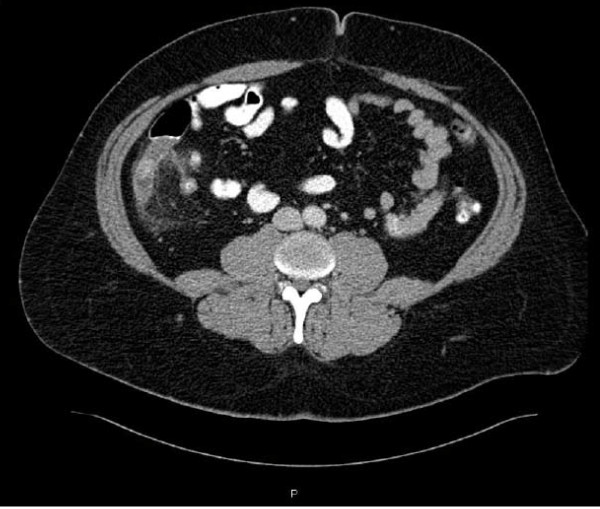
**Computed Tomography image showing the enlarged and inflamed appendix**.

The patient is a 38 year old obese male, otherwise healthy, who presented with a 24 hour history of right lower quadrant pain and anorexia. His laboratory workup revealed a leukocytosis with eighty percent neutrophilia. On abdominal examination, the patient had localized tenderness lateral to McBurney's point with a positive psoas sign. A computed tomography scan confirmed the presence of a 16 mm enlarged appendix with signs of surrounding inflammation [Figure 1]. The patient was promptly taken to the operating room for a LA. A 12 mm periumbilical trocar was placed under direct vision followed by placement of a 5 mm suprapubic port and a 5 mm left lower quadrant port. The peritoneal cavity was insufflated with carbon dioxide to a pressure of 15 mm Hg. Upon exploration of the abdomen, the appendix was confirmed to be retrocolic in location, significantly inflamed, and adherent to the posterolateral abdominal wall. As the appendix was bluntly mobilized and freed from its posterolateral attachment, a sudden small amount of venous bleeding was noted to originate behind the cecum. After the appendectomy was completed in the usual manner using two endo-GIA™ stapler loads, we focused our attention on identifying and controlling the bleeding. Upon close inspection, both staple lines appeared intact, and the bleeding was confirmed to be retroperitoneal in location, and more significant in severity than initially suspected. Repetitive attempts to expose and identify the bleeding vessel laparoscopically failed. At this point, we proceeded with a transverse Rocky-Davis muscle-splitting open incision. A Bookwalter retractor was placed, and exposure was ultimately achieved despite the patient's large body habitus (body mass index = 42 kg/m^2^). The bleeding vessel was identified as the right gonadal vein which had apparently avulsed upon mobilization of the retrocolic appendix. The testicular vein was suture-ligated with 3-0 vicryl sutures with cessation of the bleeding. Care was taken to avoid injuring the ureter. By the end of the procedure, the patient had lost 1200 ml of blood and had received two units of packed red blood cells. The patient did well after the procedure and was discharged home on the second postoperative day in stable condition without any major sequelae.

## Discussion

After almost two centuries of performing appendectomies, surgeons started resecting the inflamed appendix laparoscopically in the late 1980's. Whether the laparoscopic approach is superior, equivalent or inferior to the open approach in terms of outcomes remains controversial. Several trials have consistently showed that LA, despite being associated with a longer operative time, provides patients with a faster recovery and earlier return to routine activities when compared to OA [1-6]. In a systematic review of randomized trials conducted by Sauerland et al, the rate of superficial surgical site infection was decreased by half, but the rate of deep surgical site infections (intra-abdominal abscesses) was three times higher in LA as compared to OA [5]. On the other hand, a more recent study that used the Nationwide Inpatient Sample database from 2000 to 2005 suggested that the overall rate of complications is 7% higher with LA [9]. This same study of more than 132,000 appendectomies also found that the cost of LA was 22% higher than OA in uncomplicated appendicitis and 9% higher in complicated appendicitis. More importantly, laparoscopy has been associated with a 0.1 to 1% risk of intra-abdominal or retroperitoneal injuries, including major vessel injury [10-12]. Most of these injuries have been reported to occur during the initial trocar or Veress needle insertion, and many resulted in major morbidity to the patient. Whether LA or OA is the "standard" treatment for acute appendicitis remains controversial, and resolving that matter will probably require rigorous valuation (assigning "values" to the severity of specific complications) and severity weighting of the complication profile of each approach in the setting of a randomized trial [13].

The appendix is reported to be "hidden" in a retroperitoneal, retroileal, retrocecal or retrocolic location in up to 30% of cases [14]. The terms retrocecal, retroperitoneal and retrocolic have been and continue to be used in literature interchangeably. However, in a 1938 report, William B. Marbury defined retrocecal as being limited by the caput cecum and retrocolic as extending superiorly posterior to the ascending colon [15]. Most retrocolic appendices are also retroperitoneal, while most retrocecal appendices are intraperitoneal.

The patient we report in this paper had a major vascular retroperitoneal injury resulting in significant hemorrhage. The injury likely resulted from avulsion of the retroperitoneal gonadal vessel during dissection of the inflamed retrocolic appendix rather than from a trocar or Veress needle insertion. Marbury, in his landmark 1938 paper, reported on one patient with a retrocolic appendix who suffered "troublesome" bleeding subsequent to injury to a branch of the ileocecal artery [15]. To the best of our knowledge and following review of the literature, major bleeding during routine blunt dissection of the appendix is very rare but poses potentially significant harm to patients. We recommend that surgeons continue with meticulous dissection of any suspected retroperitoneal or retrocolic appendix. The use of advanced bipolar devices (e.g. Ligasure ™) or ultrasonic desiccation instruments (e.g. harmonic scalpel ™) might be of assistance if the appendix is severely inflamed. In addition, conversion to OA should be seriously considered when the patient shows signs of hemodynamic instability or when laparoscopic hemostatic methods fail to adequately expose and control the hemorrhage.

## Competing interests

The authors declare that they have no competing interests.

## Authors' contributions

HK, JD and RG participated in the care of the patient, including the operative part.

HK, JD and RG envisioned the concept of the manuscript.

HK wrote the first draft of the manuscript

JD and RG critically reviewed the manuscript.

HK, JD and RG all read and approved the final manuscript.
